# The potential therapeutic roles of dental pulp stem cells in spinal cord injury

**DOI:** 10.3389/fmolb.2024.1363838

**Published:** 2024-04-29

**Authors:** Jing Fu, Wenjie Li, Tengfei Mao, Zaipeng Chen, Lili Lai, Jiachen Lin, Zhiqiang Nie, Yunkai Sun, Yanqin Chen, Qin Zhang, Xigong Li

**Affiliations:** ^1^ Department of Stomatology, Hangzhou Xixi Hospital Affiliated to Zhejiang Chinese Medical University, Hangzhou, China; ^2^ Department of Anesthesiology and Surgery, Qingdao Municipal Hospital Group, Qingdao, China; ^3^ Yuncheng Central Hospital Affiliated to Shanxi Medical University, Yuncheng, China; ^4^ College of Pharmacy, Guizhou Medical University, Guiyang, China; ^5^ Department of Orthopedics, The First Affiliated Hospital, College of Medicine, Zhejiang University, Hangzhou, China; ^6^ The Eighth Clinical Medical College of Shanxi Medical University, Yuncheng, China

**Keywords:** cell therapy, human dental stem cells, mesenchymal stem cells, spinal cord injury, the potential therapeutic roles

## Abstract

Spinal cord injury (SCI) can lead to serious functional disorders, which have serious impacts on patients and society. The current traditional treatments of SCI are not effective the injured spinal cord is difficult to repair and regenerate. In recent years, stem cell transplantation for the treatment of SCI has been a hot research topic. Dental pulp stem cells have strong abilities of self-renewal and multi-directional differentiation, and have been applied for tissue engineering and regenerative medicine. And dental pulp stem cells have certain advantages in neuro-regenetation, bringing new hope to biotherapy for SCI. This article reviews the characteristics of dental pulp stem cells and their research progress in the treatment of SCI.

## 1 Introduction

Spinal cord injury (SCI) is one of the most devastating of traumatic events, which can lead to irreversible motor and sensory dysfunction below the lesion site (McDonald and Sadowsky, 2002; Eli et al., 2021). Generally, the pathophysiology of SCI usually consists of the primary injury and secondary injuries (McDonald and Sadowsky, 2002). The primary injury mainly refers to the mechanical injury involving falls from height, sports and traffic accidents. Following the primary injury, several pathological changes occur in the secondary injury, including hemorrhage, edema, demyelination, axonal and neuronal necrosis and inflammatory reaction, which primarily correlate with patient’s morbidity and mortality (Rowland et al., 2008; Ahuja and Fehlings, 2016). Therefore, the current neuroprotective and regenerative strategies mainly targeting the secondary injury are expected to be used as therapy for SCI (McDonald and Sadowsky, 2002; Rowland et al., 2008; Ahuja and Fehlings, 2016).

In recent years, biological regenerative therapy with stem cells has attracted more attention in the treatment of SCI (Bonaventura et al., 2020; Andrzejewska et al., 2021; Zipser et al., 2022). Previous experimental studies demonstrated that in animal models of SCI, stem cell therapy can improve motor and sensory functions through multifarious mechanisms, involving restoration of cell population, paracrine action, and microenvironment modulation (Bonaventura et al., 2020; Andrzejewska et al., 2021). Therefore, stem cell therapy is considered as the most promising regenerative treatment of SCI.

Mesenchymal stem cells (MSCs) have been extensively studied for biological regenerative medicine. MSCs can be sourced from a variety of tissues and organs, including bone marrow, adipose tissue, umbilical cord, blood, skeletal muscle, and oral cavity (Bonaventura et al., 2020; Andrzejewska et al., 2021). Oral-derived mesenchymal stem cells have attracted growing attention in the regenerative field based on their unique features including multipotency, easy accessibility, genomic stability, and faster proliferation rates (Bianco et al., 2016; Xu et al., 2019; Bonaventura et al., 2020; Luzuriaga et al., 2021). Dental pup stem cell (DPSC) has remained as the most extensively studied subtype of oral-derived mesenchymal stem cells (Bianco et al., 2016; Xu et al., 2019; Bonaventura et al., 2020; Bar et al., 2021; Luzuriaga et al., 2021; Mattei and Monache, 2023). Derived from the neural crest, DPSCs exhibit notable neuroregenerative potential, neurotrophic effects, and immunomodulation, which indicate that DPSCs is an ideal cell source for the injured spinal cord regeneration (Bianco et al., 2016; Xu et al., 2019; Bonaventura et al., 2020; Bar et al., 2021; Luzuriaga et al., 2021; Mattei and Monache, 2023). In this review, we will summarize the biological characteristics of DPSCs and the recent progress for the application of DPSCs in SCI treatment, with a focus on their possible regenerative mechanisms for future application in DPSC-based therapy.

## 2 The pathophysiology of the secondary injury following SCI

**FIGURE 1:**
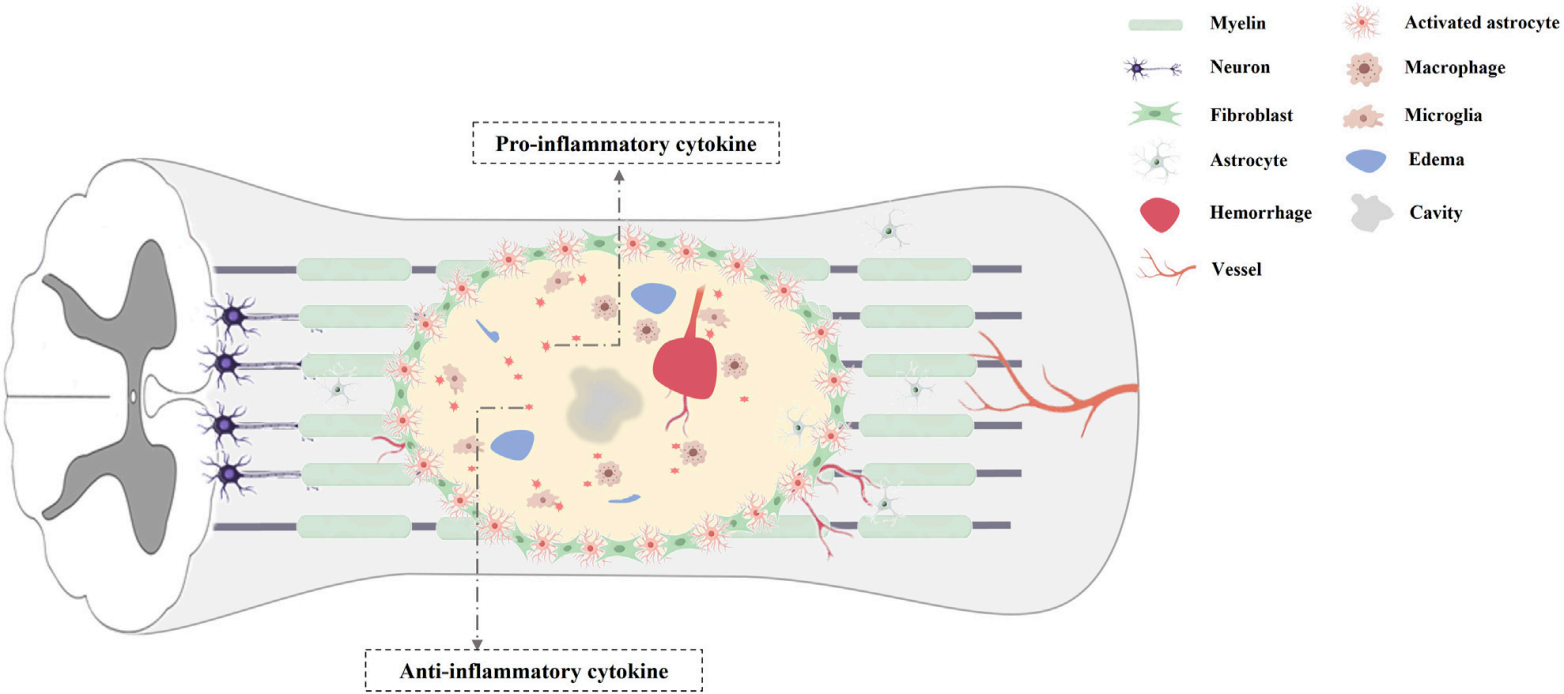
Pathological characteristics of spinal cord injury.

The secondary damage of SCI mainly involved cell death, inflammatory response, axonal collapse and demyelination, glial scar formation (Figure 1). In the acute phase of SCI, a primary mechanical damage disrupts spinal cord tissue homeostasis, which activates resident microglia and recruit macrophages from the bloodstream to the lesion site (Schwab et al., 2006; Venkatesh et al., 2019). These inflammatory cells secret multiple neurotoxic factors that induce necrotic and apoptotic death in neurons, astrocytes, and oligodendrocytes (Dumont et al., 2001; Kwon et al., 2004; Fan et al., 2018; Venkatesh et al., 2019). And the initial mechanical injury, ischemia, inflammatory response can cause irreversible axonal damage, and the necrosis and apoptosis of oligodendrocytes, which eventually results in the process of demyelination (Dumont et al., 2001; Kwon et al., 2004; Fan et al., 2018; Venkatesh et al., 2019). In addition, reactive astrocytes and oligodendrocytes near the injured spinal cord center generate chondroitin sulfate proteoglycans and myelin proteins, which can cause growth cone collapse, neurite retraction and inhibition of axon regeneration (Siebert et al., 2014). Furthermore, the chondroitin sulfate proteoglycans generated by activated microglia, macrophages and astrocytes, can form a glial scar that also inhibits spontaneous axonal regeneration (Ohtake and Li, 2015). Finally, multiple pathogenic signals synergistically accelerate progressive neuronal deterioration after SCI. Thus, an ideal biological therapy for SCI not only can markedly reduce secondary damage, but also replace the damaged neuron, axons, and circuits within the spinal cord. Transplantation of stem cells has been applied for biological treatment in animal models of SCI, with favorable functional recovery (Ahuja and Fehlings, 2016; Andrzejewska et al., 2021; Zipser et al., 2022). Despite the biological characteristics of various types of stem cells differ, the therapeutic benefits of stem cells in SCI have been reported including replacing lost cells, modulating inflammatory reaction, improving the microenvironment and promoting regeneration (Zipser et al., 2022).

## 3 Biological characteristics of DPSCs

**FIGURE 2:**
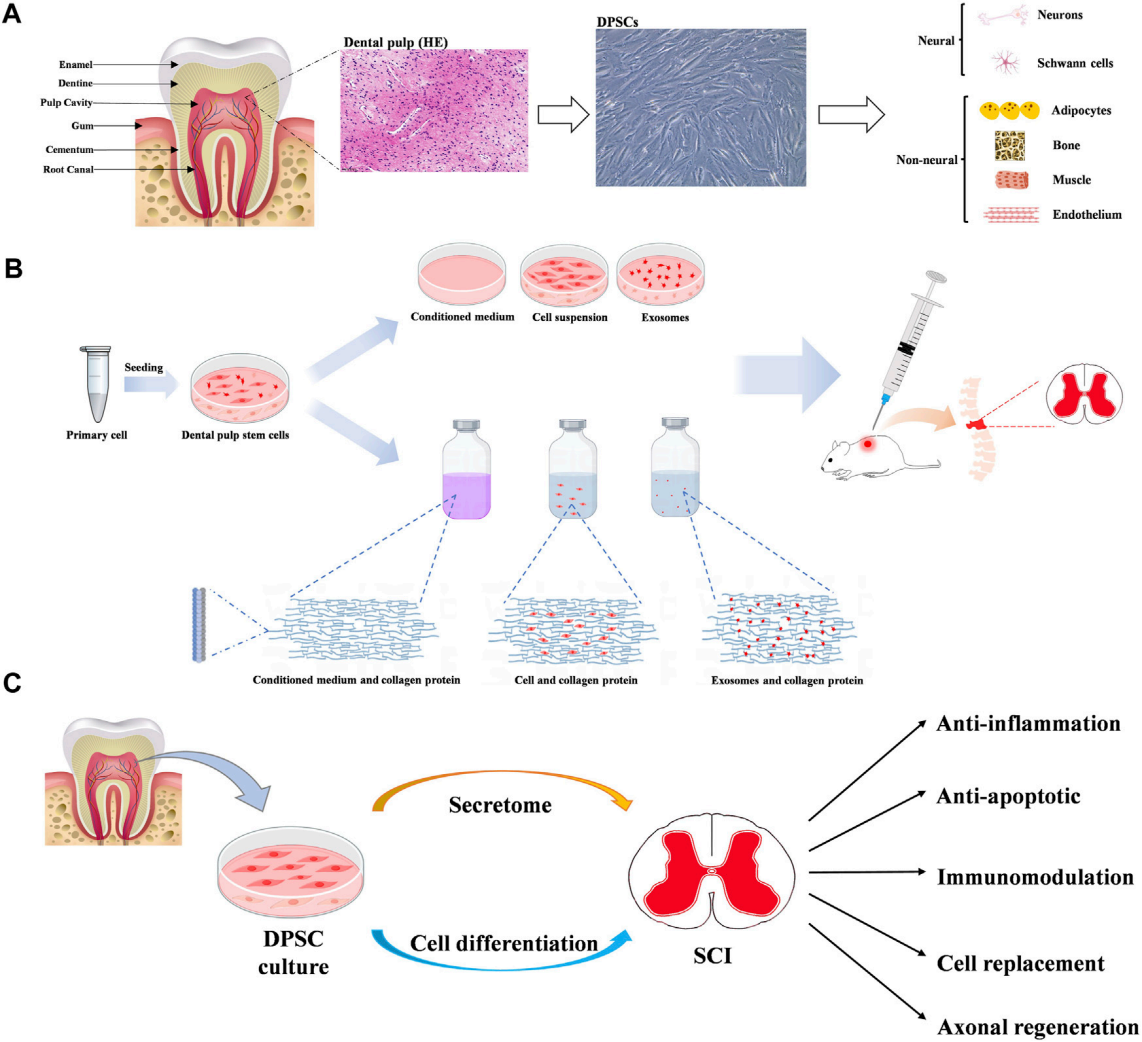
**(A)** Diagram of tooth, dental pulp and dental pulp stem cells (DPSCs). And multiple differentiation potentials of DPSCs into various cell types. **(B)** The main mechanisms of dental pulp stem cells in neuroprotection. **(C)** The potential benefits of dental pulp stem cells in therapy for spinal cord injury.

DPSCs deriving from child or adult human dental pulp were initially discovered by Gronthos and colleagues in 2000 (Gronthos et al., 2000). The dental pulp tissue generated by the neural crest, is rich in odontoblasts, blood vessels, nerve fibres, immune cells and mesenchymal stem cells. DPSCs isolated from dental pulp, can express neural markers, including glial fibrillary acidic protein (GFAP), β-III tubulin, and microtubule-associated protein-2 (MAP-2) (Martens et al., 2012; Li et al., 2019). In addition, DPSCs have been found to express mesenchymal-like phenotypic markers (CD29, CD90, and CD73) (Li et al., 2019), express stemness-related markers (Oct-4, Nanog, and Sox-2) (Kerkis et al., 2006), cytoskeleton-related markers (Nestin and Vimentin), tumor necrosis factor receptor superfamily proteins (CD40, CD120a, CD261, CD262, CD264 and CD266), some integrins (alpha-4, alpha-6 and alpha-10) and IL receptors (CD121a, CD130, CD213a1, CD217 and CDw210b) (Niehage et al., 2016). Recently, several new special markers are identified, such as Calreticulin, Annexin A5, and Rho GDP dissociation inhibitor alpha (Lei et al., 2021). Isolated DPSCs can not only maintain and repair periodontal tissue, with the feature of high proliferation rate, but also show plasticity in multi-lineage differentiation. Several *in vitro* studies have confirmed DPSCs have the potential to differentiate into multiple cell types such as osteoblasts-, chondrocytes-, adipocytes-, odontoblasts-, neural- and myocytes-like cells (Fu et al., 2023) (Figure 2).

Furthermore, DPSCs have some biological advantages over other tissue-derived MSCs. Because the dental pulp has similar structure like neurovascular tissue, DPSCs have very higher ability to generate neural and vascular cells than other MSCs (Luzuriaga et al., 2020; Fu et al., 2023). And It has been demonstrated the secretion of nerve growth factor (NGF) and brain derived neurotrophic factor (BDNF) by DPSCs are very higher than bone mesenchymal stem cells. (McDonald and Sadowsky, 2002; Eli et al., 2021). The secrecion of vascular endothelial growth factor-A and vascular endothelial growth factor-D by DPSCs are higher than other MSCs (Rowland et al., 2008; Ahuja and Fehlings, 2016). These results indicate DPSCs have very higher capacity for angiogenesis and neurogenesis.

## 4 The potential mechanisms of DPSCs in SCI

Previous studies have demonstrated DPSCs show the potential therapies for SCI through multiple mechanisms, mainly involving neuronal differentiation, paracrine effects and exosome secretion (Xu et al., 2019; Bonaventura et al., 2020; Fu et al., 2023) (Figure 2).

### 4.1 Neuronal-like differentiation from hDPSCs

DPSCs retain the properties of neural crest cells, which possess the differentiation capacity into neural crest-derived tissue. Several neural markers can be expressed in non-differentiated DPSCs, such as musashi12, nestin, MAP2ab, βIII-tubulin, N-tubulin, and neurogenin-2 (Kawashima, 2012; Luzuriaga et al., 2019a). Thus, DPSCs have been promoted as potential candidates for damaged neuron replacements. Previous studies have demonstrated DPSCs not only can express several typical neural stem/progenitor markers under the specific stimulation, but also differentiate toward phenotypes of functionally active neurons, with the higher expression of neurogenin-2, neuron-specific enolase, neurofilament-M and glial fibrillary acidic protein, and activation of voltage-gated sodium and potassium channels (Luzuriaga et al., 2021). Multiple stimulations have been used to differentiate DPSCs into neuron-like cells, involving several differentiating protocols containing complex mixture of supplements (Luzuriaga et al., 2019b), optogenetics (Niyazi et al., 2020), activation of K+ channels (Kogo et al., 2020), and Rho kinase inhibitor (Srikawnawan et al., 2022). Darvishi et al. demonstrated human DPSCs can differentiate to functional motor neuron in organotypic culture system containing growth factors in two stages (Darvishi et al., 2021). Differentiated DPSCs not only display motor neuron-like morphology, with Nissl bodies in the cytoplasm, but also express several motor neuron specific genes in post-induction stage, such as Olig2, Islet-1 and HB9. In animal models of SCI, transplantation of dental-derived mesenchymal stem cell alone or as part of designed engineered tissue also have been shown to differentiate neuron-like phenotype, replacing the defective neuronal tissue and improving functional recovery after SCI (Zhang et al., 2016; Yang et al., 2017; Kabatas et al., 2018; Qian et al., 2023; Zhou et al., 2023). Recently, Qian et al. found the DPSC-loaded microspheres have improved stemness and higher neurogenic differentiation potential of DPSCs, promoting neural tissue regeneration in rat models of SCI (Qian et al., 2023). The combination of biomaterials with DPSCs represents a promising approach to enhance neurogenic differentiation of DPSCs. The combination of chitosan 3D porous scaffolds with bFGF enhances the expression of neural markers in DPSCs through the activation of the extracellular signal-regulated kinase (ERK) pathway (Zhang et al., 2016). Moreover, usage of the DPSC-chitosan grafts improve locomotor function in animal models of SCI, by the secretion of BDNF, GDNF and NT-3, reducing the accumulation of active-caspase 3, and impairing axonal loss and degradation. Zhou et al. reported the roles of bioactive material zeolitic imidazolate framework 8 (ZIF-8) on neural differentiation of DPSCs (Zhou et al., 2023). These authors found after injection of methacryloyl hydrogel containing ZIF-8-introduced DPSCs into the lesion, axon number and axon length of DPSCs-differentiated neuron-like cells were significantly increased. ZIF-8 can promote neural differentiation and angiogenesis of DPSCs by activating the mitogen-activated protein kinase signaling pathway. In addition, DPSCs can induce the formation of extensive axonal regeneration and establish close contacts with cocultured neurons (Pagella et al., 2020), which indicates functional synapses may be formed. All mentioned above indicate DPSCs can display a capacity for neuronal differentiation both *in vivo* and *in vitro*.

### 4.2 Paracrine effects

The paracrine effects of DPSCs have been highlighted in regenerative medicine. It has been reported that DPSCs can secrete many bioactive molecules such as interleukin (IL)-10, IL-13, follistatin, transforming growth factor-β1, hepatocyte growth factor, neural cell adhesion molecule-1, and adiponectin (Ogata et al., 2021; Ogata et al., 2022). The bioactive molecules secreted by DPSCs can directly affect their own intracellular signal transduction, and can also indirectly cause neighboring cells to secrete functional active substances (Mattei et al., 2021). Moreover, several neuroprotective factors can be detected in DPSC-conditioned medium (DPSC-CM), including nerve growth factor (NGF), brain-derived neurotrophic factor (BDNF), glial cell line derived neurotrophic factor (GDNF), neurotrophin 3 (NT-3), vascular endothelial growth factor (VEGF) and platelet-derived growth factor (PDGF) (Matsumura-Kawashima et al., 2021; Bousnaki et al., 2022; Li et al., 2023). Asadi-Golshan et al. found intraspinal administration of DPSC-CMs loaded in collagen hydrogel can dramatically improve functional recovery in rat SCI model (Asadi-Golshan et al., 2018). And the total loss number of neurons and oligodendrocytes, and the spinal cord lesion volume were significantly decreased after DPSC-CM therapy, which indicated DPSC-CM containing growth factors are able to promote axon regeneration and neuron survival in the early stages of SCI. Moreover, de Almeida et al. found DPSCs transplanted in a rat model of SCI could release trophic factors into the damaged spinal cord tissue, inducing the presence of axons, expressing some glial markers and improving locomotor functions (Asadi-Golshan et al., 2021). Furthermore, DPSCs can secrete several immunomodulatory and anti-inflammatory cytokines such as Interleukin-8 (IL-8), Interleukin-6 (IL-6), Transforming Growth Factor Beta (TGF-β), Hepatocyte Growth Factor (HGF) and Indoleamine 2,3-dioxygenase (IDO) (Mattei et al., 2021). Matsubara et al. show that DPSC-CM containing EDSiglec-9 and monocyte chemoattractant protein-1can induce significant functional recovery in a rodent SCI model by promoting polarization of M2 macrophages (Kano et al., 2017). In addition, intraperitoneally injected-DPSC-CM effectively decreased the microglial pyroptosis by inhibiting the NLRP3/caspase-1/interleukin-1β pathway, thereby promoting the functional recovery after SCI (Liu et al., 2024). Furthermore, DPSCs can secrete several immunomodulatory and anti-inflammatory cytokines such as Interleukin-8, Interleukin-6, Transforming Growth Factor Beta, Hepatocyte Growth Factor and Indoleamine 2,3-dioxygenase (Yamamoto et al., 2014). Matsubara et al. show that DPSC-CM containing EDSiglec-9 and monocyte chemoattractant protein-1 can induce significant functional recovery in a rodent SCI model by promoting polarization of M2 macrophages (Asadi-Golshan et al., 2021). In addition, intraperitoneally injected- DPSC-CM effectively decreased the microglial pyroptosis by inhibiting the NLRP3/caspase-1/interleukin-1β pathway, thereby promoting the functional recovery after SCI (Kano et al., 2017). Furthermore, several bioactive factors secreted from DPSCs can also promote the regeneration of transected axons, through directly inhibiting multiple axon growth inhibitors, including chondroitin sulfate proteoglycan and myelin-associated glycoprotein (Sakai et al., 2012). Therefore, the paracrine effects of DPSCs, mainly consisting of trophic support and immunomodulation, can be used specifically in the repair of damaged spinal cord tissue. Therefore, the paracrine effects of DPSCs, mainly consisting of trophic support and immunomodulation, can be used specifically in the repair of damaged spinal cord tissue.

### 4.3 DPSCs-exosomes in SCI

MSCs can secret multiple molecules as soluble factors and extracellular vesicles, which are involved in the transference of various proteins and genetic materials (Hade et al., 2021). Exosomes (EXs), one small particule of extracellular vesicles (usually 40–120 nm in diameter), containing many bioactive macromolecules, such as proteins, nucleic acids and lipids involved in biological regulation of cells (Liu et al., 2021). Several previous studies demonstrated MSC-derived EXs have the ability to enhance functional recovery in animal model of SCI by reducing cell apoptosis and inflammation response, and promoting angiogenesis (Liu et al., 2021; Mohebichamkhorami et al., 2022). Depending on the cell sources, MSC-derived EXs have specific set of proteins and nucleic acids that can promote tissue regeneration. Currently, Oral-derived EXs have gained more attention due to their potentials in therapy for SCI, which have more biological regulatory property in anti-inflammation, immunomodulatory and neuroprotection (Lambrichts et al., 2017; Li et al., 2021; Li et al., 2022; Liu et al., 2022). Periodontal ligament-derived EXs in SCI models lead to decrease in pro-inflammatory CD4-positive T cells and inflammation cytokine expression such as IL-6, IL-17, IL-1β, IFN-γ, and TNF-α (Li et al., 2021). Jonavice et al. showed that DPSC-EVs can alter the phenotypes of microglia by suppressing NF-κB signalling pathway. The shifting microglia M1/M2 polarization can eventually contribute to improve microenvironment and functional recovery after traumatic brain injury (Li et al., 2022). Recently, Liu et al. demonstrate that administration of DPSCs-EXs can decrease the inflammatory response and minimize neurological impairment by reducing macrophage M1 polarization through suppressing ROS-MAPK-NFkB P65 signaling pathway (Liu et al., 2022). In addition, DPSC-EXs play important roles in neuronal axon regeneration, which indicated their potential therapies for neurite growth, and axon remodeling of spinal cord tissue (Lambrichts et al., 2017). Therefore, DPSC-EXs as biological extracellular vesicles can promote functional recovery in SCI through multiple neuroprotecitve mechanisms.

## 5 Conclusion

Previous published literature demonstrate DPSCs represent a promising new cell source for cell-based treatment of SCI, with the evidence of three mainly effective patterns, including neuronal differentiation, paracrine effects and exosome secretion (Table 1). Transplant of DPSCs or DPSCs-secretome can provide several neuroprotective benefits for the treatment of SCI, such as suppressing inflammatory response, inhibiting SCI-induced cellular apoptosis, modulating promoting axonal regeneration, and cell replacement especially in providing functional neuronal-like cell (Figure 2). These regenerative mechanisms have shown great potential in reconstructing the injured spinal cord and promoting functional recovery in experimental model of SCI. However, many problems remain to be resolved before the clinical usage of this therapy. Firstly, it is preliminary important to achieve large-scale DPSCs manufacture, storage, and transportation with minimum possibility of contamination. In addition, concerns remain raised about the biological safety of DPSCs therapy such as immunotoxicity, immunogenicity, and tumorigenicity. Moreover, the quality of DPSCs is critical to clinical usage, such as the standard source of dental pulp and culture conditions. Therefore, future researches should be further investigated to guide the clinical application of DPSCs.

**TABLE 1:** Studies that assessed the use of dental pulp stem cells for spinal cord repair and regeneration.

Dental stem cells (DSCs)/scaffold	Animal model	Remarks	Year	References
Dental pulp stem cells (DPSCs)	Mouse model of Compressive spinal cord injury	DPSCs induce neuroplasticity and endogenous axon guidance in a mouse model of SCI	2011	de Almeida et al. (2011)
Dental pulp stem cells (DPSCs); exfoliated deciduous teeth stem cells (SHEDs)	Injured rat spinal cord	DPSCs and SHEDs possessed higher neuroregenerative activities than BMSCs and provided significant benefits for SCI; DSCs can ameliorate several aspects of functional recovery after SCI	2012	Sakai et al. (2012)
Dental pulp stem cells (DPSCs) and chitosan scaffolds	SCI rat model	Transplantation of DPSCs together with chitosan scaffolds into an SCI rat model resulted in the marked recovery of hind limb locomotor functions	2016	Zhang et al. (2016)
Human dental follicle stem cells (DFSCs), stem cells from apical papilla (SCAPs) dental pulp stem cells (DPSCs)	SCI rat model	DFSCs, demonstrated the potential in repairing the completely Transected spinal cord and promote functional recovery after injury	2017	Yang et al. (2017)
Thermosensitive heparin-poloxamer (HP) hydrogel containing DPSCs and bFGF	Injured rat spinal cord	Hydrogel containing DPSCs and bFGF had a significant impact on spinal cord repair and regeneration	2018	Luo et al. (2018)
fibroblast growth factor (FGF) 2-pretreated human dental pulp cells (hDPCs)	SCI rat model	the role of FGF2-responsive genes, especially GABRB1, in recovery from SCI, using hDPCs treated with FGF2	2019	Sugiyama et al. (2019)
Fibroblast growth factor (bFGF) and dental pulp stem cells (DPSCs)	SCI mouse model	bFGF and DPSCs worked together to attenuate tissue inflammation of the injured spinal cord, resulting in a superior nerve repair	2020	Albashari et al. (2020)
Highly vascularized scaffolds embedded with human dental pulp stem cells (DPSCs)	a rat complete spinal cord transection model	prevascularized DPSC-embedded constructs bear angiogenic and neurotrophic potentials, capable of augmenting and modulating SCI repair	2020	Guo et al. (2020)
A calcium alginate hydrogel combined with dental pulp stem cells (DPSCs) and fibroblast growth factor 21 (FGF21)	mice model of HSCI	Ca2+@Alg-FGF21 + DPSC hydrogel could effectively promote the recovery after spinal cord hemisection in mice via regulating apoptosis and autophagy	2021	Zhu et al. (2021)
Human dental pulp stem cells (hDPSCs) and platelet-rich plasma (PRP)	SCI rat model	Significantly increased inhibition of neuronal apoptosis and improved motor function recovery of the spinal cord were observed following double-treatment with hDPSCs and PRP	2022	Hu et al. (2022)
Dental pulp stem cell (DPSC)-derived exosomes	SCI rat model	Dental pulp stem cell (DPSC)-derived exosomes can reduce macrophage M1 polarization through the ROS-MAPK-NFκB P65 signaling pathway in treating SCI.	2022	Liu et al. (2022)
Human dental pulp stem cells (hDPSC) cultivated in monolayer (2D) or as spheroids (3D)	SCI rat model	the 2D and 3D cell therapy approaches provide successful immunomodulation and motor recovery	2023	Paes et al. (2023)
Human dental pulp stem cells (hDPSC)-loaded microspheres	SCI rat model	hDPSC-loaded microspheres could promote spinal cord regeneration in rat spinal cord injury models	2023	Qian et al. (2023)
Dental pulp stem cells (DPSCs) were introduced into the TPA@Laponite hydrogel	SCI rat model	effectively reduced muscle spasms and promoted recovery from SCI	2023	Ying et al. (2023)
Dental pulp stem cells (DPSCs) and zeolitic imidazolate framework 8 (ZIF-8)	SCI rat model	ZIF-8 promotes neural differentiation and angiogenesis of DPSCs by activating the Mitogen-activated protein kinase (MAPK) signaling pathway	2023	Zhou et al. (2023)
Conditioned medium from human dental pulp stem cells (hDPSCs)	a rat model of spinal cord injury	conditioned medium from human dental pulp stem cells can reduce microglial pyroptosis by inhibiting the NLRP3/caspase-1/interleukin-1β pathway, thereby promoting the recovery of neurological function after spinal cord injury	2024	Liu et al. (2024)
